# FAK activity protects nucleostemin in facilitating breast cancer spheroid and tumor growth

**DOI:** 10.1186/s13058-015-0551-x

**Published:** 2015-03-28

**Authors:** Isabelle Tancioni, Nichol LG Miller, Sean Uryu, Christine Lawson, Christine Jean, Xiao Lei Chen, Elizabeth G Kleinschmidt, David D Schlaepfer

**Affiliations:** 1 Department of Reproductive Medicine, University of California San Diego, Moores Cancer Center, 3855 Health Sciences Dr, La Jolla, CA 92093 USA; 2 Current address: Pfizer, La Jolla, CA 92121 USA; 3 Current address: INSERM U1037 - Cancer Research Center, Toulouse, France

## Abstract

**Introduction:**

Focal adhesion kinase (FAK) controls cell growth and survival downstream of integrin-matrix receptors. Upon adhesion loss or FAK inhibition, FAK can translocate to the nucleus. The nucleolus is a non-membrane nuclear structure that regulates ribosome biogenesis and cell proliferation. Nucleostemin (NS), a nucleolar-localized protein, modulates cell cycle progression, stemness, and three-dimensional tumor spheroid formation. The signaling pathways that regulate NS levels in tumors remain undefined.

**Methods:**

Human breast carcinoma cells were evaluated for growth in culture (adherent and anchorage-independent spheroid) and as orthotopic tumors. FAK signaling was evaluated by pharmacological FAK inhibitor addition (PF-271, IC50 ~ 0.1 μM) and by small hairpin RNA (shRNA) knockdown followed by re-expression of FAK wildtype (WT) or a kinase-dead (KD, K454R) FAK point mutant. Immunoblotting was used to evaluate FAK, NS, nucleolar phosphoprotein B23, and nucleolin levels. Total and phosphospecific antibody imunoblotting were used to detect changes in FAK, Akt kinase (Akt also known as protein kinase B), and 4E-binding protein 1 (4E-BP1) phosphorylation, a translation repressor protein and target of the mammalian target of rapamycin (mTOR) complex. Immunohistochemical, co-immunoprecipitation, and cellular fractionation analyses were used to evaluate FAK association with nucleoli.

**Results:**

Pharmacological (0.1 μM PF-271) or genetic inhibition of FAK activity prevents MDA-MB-231 and 4T1L breast carcinoma growth as spheroids and as orthotopic tumors. FAK inhibition triggers proteasome-mediated decreased NS levels but no changes in other nucleolar proteins such as B23 (nucleophosmin) or nucleolin. Active FAK was associated with purified nucleoli of anchorage-independent cells and present within nucleoli of human invasive ductal carcinoma tumor samples. FAK co-immunoprecipitated with B23 that binds NS and a complex between FAK, NS, Akt, and mTOR was detected. Constitutively-active Akt kinase promoted tumor spheroid growth, stabilized NS levels, and promoted pS65 4E-BP1 phosphorylation in the presence of inhibited FAK. Rapamycin lowered NS levels and inhibited pS65 4E-BP1 phosphorylation in cells with activated Akt-mTOR signaling.

**Conclusions:**

FAK signaling occurs in the nucleolus, active FAK protects NS, and Akt-mTOR pathway regulates NS protein stability needed for breast carcinoma spheroid and tumor growth.

**Electronic supplementary material:**

The online version of this article (doi:10.1186/s13058-015-0551-x) contains supplementary material, which is available to authorized users.

## Introduction

Breast cancer is one of the most common cancers in women worldwide [1]. It is a heterogeneous disease with differential responses to therapy [2]. Triple-negative breast cancers exhibit resistance to various chemotherapies and are the most aggressive tumors with a 5-year survival rate of <30% [3]. Relapse and patient mortality results in part from tumor spread and metastasis [4].

Signals generated from transmembrane integrin receptors are one of the molecular drivers of tumor metastasis [5]. Integrins sense changes in extracellular matrix composition and tension and in turn activate focal adhesion kinase (FAK), a 115 kDa cytoplasmic tyrosine kinase [6]. FAK mRNA levels are elevated in approximately 26% of breast tumors, and high FAK protein levels are common in human epidermal growth factor 2 (HER2)-positive [7] and triple-negative tumors [8]. FAK overexpression is associated with increased tumor growth, an invasive phenotype, higher histological grade, and poor patient prognosis [8-10]. Mouse tumor models reveal that FAK knockout prevents multiple aspects of breast carcinoma tumor initiation and progression [11-14].

Studies evaluating genetic or pharmacological inactivation of FAK activity within tumor cells have linked FAK signaling to the promotion of tumor growth, angiogenesis, and tumor metastasis [6,15]. *In vitro*, sub-micromolar concentrations of pharmacological FAK inhibitors can prevent tumor cell growth under three-dimensional but not necessarily two-dimensional conditions [16-19]. This is associated with tumor cells expressing low merlin tumor suppressor expression and β1 and β5 integrin signaling linkages [19-21]. Tumor spheroid survival is highly dependent on FAK activity [16,21]. These FAK-associated signals can be generated at the cell surface in association with integrins. However, because FAK also translocates to the nucleus when cells are grown in suspension [22], and nuclear FAK can alter gene expression [23,24], the location for FAK-mediated survival signal generation within tumor spheroids remains undefined.

It also remains unclear whether FAK activity within a tumor spheroid impacts the expression of target proteins selectively required for three-dimensional or anchorage-independent cell survival. To this end, tumor spheroids share many characteristics of cancer stem cells [25]. Several proteins associated with the nucleolus play important roles in genome protection, ribosome synthesis, and stem cell proliferation [26,27]. Nucleostemin (NS), a 62-kDa nucleolar protein, regulates proliferation in stem and cancer cells [28,29]. Interestingly, NS promotes anchorage-independent mammary tumor spheroid growth [30] and is increased during mouse mammary tumor progression [31], and breast cancer patients with NS-positive tumors exhibit significantly shorter disease-free survival [32]. NS is a guanine triphosphate (GTP)-binding protein that shuttles between the nucleolus and nucleoplasm [33]. Inhibition of guanine nucleotide synthesis triggers nucleolar stress, NS movement to the nucleoplasm, and NS proteasomal degradation [34,35]. Although increased levels of reactive oxygen species can enhance NS protein stability [36], the signaling pathways controlling NS levels in tumor cells remain undefined.

Here, we show that FAK and NS are part of a signaling axis promoting breast carcinoma tumor progression. Pharmacological or genetic FAK inhibition reduces NS levels in cells grown as tumors by regulating its stability. FAK activity and NS are required for anchorage-independent spheroid growth. Cell fractionation analyses revealed active FAK association with nucleoli and a complex between FAK, NS, Akt, and mammalian target of rapamycin (mTOR) was detected by co-immunoprecipitation with green fluorescent tagged NS. Mechanistically, activated Akt kinase expression was sufficient to rescue effects of FAK inhibition and rapamycin treatment triggered loss of NS protein in cells with activated Akt-mTOR signaling. Our studies support a non-canonical role for nucleolar-associated FAK signaling and Akt-mTOR activation in the maintenance of NS protein expression.

## Methods

### Antibodies and reagents

Antibodies to pY397 FAK (clone 141–9) were from Life Technologies (Carlsbad, CA, USA). Antibodies to Akt (clone C67E7), pS473 Akt (9271), mTOR (2983) and pS65 4E-BP1 (9451) were from Cell Signaling (Beverly, MA, USA). Antibodies to NS (70346), B23 (clone FC82291), and pY397 FAK (4803) were from Abcam (Cambridge, MA, USA). Anti-human nucleolin (clone D-6) and anti-Lamin B (sc-6216) were from Santa Cruz Biotechnology (Santa Cruz, CA, USA). Monoclonal (clone 4.47) and polyclonal antibodies to FAK (06–543) were from Millipore (Bedford, MA, USA). Antibodies to β-actin (clone AC-74, Sigma) and GAPDH (clone GT239) were Sigma (St. Louis, MO, USA) and GenTex (Irvine, CA, USA), respectively. Monoclonal antibody (clone B34) and polyclonal (A-6455) to GFP and were from Covance (Princeton, NJ, USA) and Life Technologies, respectively. Plasmid pLKO.1-puro non-target shRNA control (SHC016) was from Mission-Sigma (St. Louis, MO, USA). Human NS shRNA lentivirus plasmids (in pLKO.1-puro) were from Mission-Sigma (clone IDs 206826.1-683s21c1 and 206826.1-1310s21c). Plasmid pBabe-puro and pBabe-Puro-Myr-Flag-Akt1 (15294) were from Addgene (Cambridge, MA, USA). MG132 and rapamycin were from Calbiochem (San Diego, CA, USA) and LC Laboratories (Woburn, MA, USA), respectively. PF-562,271 (PF-271) was synthesized as described [37] and PND-1186 (renamed VS-4718 by Verastem Inc.) was from Poniard (San Francisco, CA, USA). For *in vitro* studies, PF-271 and PND-1186 were dissolved in dimethyl sulfoxide (DMSO).

### Cells

The 4T1 murine mammary carcinoma cells, BT474, MDA-MB-231 and MDA-MB-468 human breast carcinoma cells were from American Type Culture Collection. MCF-7 human breast carcinoma cells were obtained from David Cheresh (UCSD, University California San Diego, CA, USA). Selection of highly metastatic mCherry 4T1 cells named 4T1L was performed by isolation and expansion of cells from lung metastases [15]. FAK shRNA-expressing HEY cells (ovarian cancer cells) were generated and cultivated as described [19]. Table 1 lists source, culture conditions, and selective DNA sequencing information for the breast carcinoma cells used in this study.

**Table 1:** **Background information on the breast carcinoma cell lines used in this study**

**Cells**	**Source**	**Culture media**	**Cancer type**	**Selected genetic events** [51]
**MCF7**	D. Cheresh (UCSD)	(1)	Invasive ductal carcinoma [52]	PIK3CA (E545K)
**BT474**	ATCC	(1)	Invasive ductal carcinoma [52]	PIK3CA (K111N)
				TP53 (E285K)
				HER2 (amplification)
**MDA-MB-231**	ATCC	(2)	Adenocarcinoma [52]	BRAF (G464V)
				KRAS (G13D)
				TP53 (R280K)
**MDA-MB-468**	ATCC	(2)	Adenocarcinoma [52]	PTEN (V85_splice)
**4T1L**	D Schlaepfer (UCSD)	(2)	Selection of metastatic mCherry-labeled 4 T1 cells by isolation and culture of cells from 4 T1 lung metastases [15]	(undetermined)

(1) Roswell Park Memorial Institute medium supplemented with 10% FBS, 0.1 mM non-essential amino acids, 2 mM glutamine, 100 U/ml penicillin, and 100 μg/ml streptomycin. (2) DMEM supplemented with 10% FBS, 0.1 mM non-essential amino acids, 2 mM glutamine, 100 U/ml penicillin, and 100 μg/ml streptomycin.

### DNA and retroviral constructs

Short-hairpin (shRNA) targeting human FAK and a scrambled (Scr) control in pLentiLox 3.7-puro were created as described [22]. Lentiviral transduced cells were selected by growth in puromycin, clones were isolated by single cell sorting, and characterized by anti-FAK immunoblotting as described [17]. Three cell clones were pooled, expanded, and stored frozen as Scr- or FAK shRNA-expressing MDA-MB-231 cells. Green fluorescent protein (GFP)-tagged murine FAK wildtype (WT) or murine FAK kinase-dead (KD, K454R) in pCDH1-MCS1-EF1-Puro (System Biosciences) were stably re-expressed by lentiviral transduction and puromycin selection. MDA-MB-231 FAK-WT and FAK-KD cells were stably transduced with a myristoylated and membrane-targeted form of Akt (Addgene) via retroviral transduction as described [22]. Cells were transduced with fluorescent mCherry protein (pmCherry-C1, Clontech) using a lentivirus expression vector (pCDH-CMV-MSC1, System Biosciences) as described [17] prior to initiation of mouse tumor studies. Human NS was amplified by PCR and subcloned into pEGFP-C1 (Clonetch) from pBabe-NS provided by Frederic Lessard and Gerardo Ferbeyre (Université de Montréal). MDA-MB-231 cells were transiently transfected using jetPRIME (PolyPlus Transfection) GFP or GFP-NS plasmid DNA (10 μg).

### Cell growth

For adherent growth, 1 × 10^4^ cells in 2 ml growth media were distributed in 6-well plates (Costar, tissue culture treated). Between 24 and 120 hours, all cells were collected by limited trypsin-EDTA treatment, a single cell suspension was prepared, and the viable total cell number determined by automated trypan blue staining and counting (ViCell XR, Beckman). For three-dimensional cultures, 10,000 cells were embedded in 1% methylcellulose diluted in growth media and plated onto 6-well poly-hyodroxyethyl methacrylic acid (poly-HEMA)-coated plates (Costar). At the indicated time, colonies were imaged in phase contrast, and enumerated by counting five different fields of each well. All experimental points were performed in triplicate and repeated at least two times.

### Mice

Female NOD/SCID (Harlan Laboratories, Indianapolis, IN, USA) mice were housed in pathogen-free conditions according to the Association for the Assessment and Accreditation for Laboratory Animal Care guidelines. Studies were performed with approval of the University of California, San Diego Institutional Animal Care and Use Committee protocols. No body weight loss or morbidity was associated with the study protocols.

### Mouse tumor studies

MDA-MB-231 (0.5 × 10^6^) cells in 10 μl of growth factor-depleted Matrigel (BD Biosciences) were injected into the T4 mammary fat pad of 8- to 10-week-old mice using a Hamilton syringe and a 28-gauge needle. Tumors were measured every 3 to 4 days with digital vernier calipers and tumor volume (mm^3^) was calculated using the formula:

$$ \mathrm{V}=\mathrm{ax}{\mathrm{b}}^2/2 $$

where V = volume, a = length, mm and b = width, mm.

Sites of spontaneous lung metastasis were imaged using the OV100 Small Animal Imaging System (Olympus) with a set exposure and quantified by combined dorsal and ventral fluorescent images using Image J (v1.46r) with a set threshold.

### Immunoblotting

Cell lysis buffer (1% Triton X-100, 1% sodium deoxycholate, 0.1% SDS, 50 mM HEPES (4-(2-hydroxyethyl)-1-piperazineethanesulfonic acid) pH 7.4, 150 mM NaCl, 10% glycerol, 1.5 mM MgCl_2_, 1 mM EGTA, 10 mM sodium pyrophosphate, 100 mM NaF, 1 mM sodium orthovanadate, and 10 μg/mL leupeptin) was used to extract proteins from cultured cells and tumors were homogenized (Pro 200 tissue homogenizer, Pro Scientific) in lysis buffer without sodium deoxycholate and SDS. Total protein levels were determined by Bradford assay (Thermo Scientific), proteins were resolved by NuPAGE 4-12% Tris-Bis gels (Invitrogen), and transferred to polyvinylidene difluoride membranes (Immobilon, Millipore) for antibody immunoblotting. Relative expression levels and phosphospecific antibody reactivity were measured by densitometry analyses of blots using Image J. For immunoprecipitation, lysates were diluted threefold in cell lysis buffer and incubated with polyclonal anti-FAK antibody or anti-GFP at 4°C, overnight. After incubation with protein A Plus agarose beads (Millipore) for 1 hour at 4°C, antibodies were collected by centrifugation, washed twice with cell lysis buffer and resolved by PAGE.

### Nucleolar isolation

Cell fractionation to isolate nucleoli was performed as described [38] with some modifications. Briefly, 0.5 × 10^7^ cells were collected by trypsinization and processed, or plated onto poly-HEMA-coated plates for 4 hours prior to processing. Cells were lysed using a hypotonic solution (10 mM HEPES pH 7.9, 10 mM KCl, 1.2 mM MgCl_2_, 0.3% NP-40 and 0.5 mM dithiothreitol) and dounce homogenized with a tight pestle. Intact nuclei were pelleted by centrifugation (228 × g for 5 minutes) and the cytoplasmic supernatant saved and stored frozen at −20°C. Nuclei were resuspended in 0.25 M sucrose, 10 mM MgCl_2_ solution (S1 solution) and layered over 3 mL 0.35 M sucrose, 0.5 mM MgCl_2_ solution (S2 solution) and centrifuged at 1430 × g for 5 minutes at 4°C. The clean, pelleted nuclei were resuspended in S2 solution and sonicated eight times (10-s bursts at 1-minute intervals) using a 450 Branson Sonifier and microtip probe at power setting 4. The sonicate was assessed by light microscopy to certify the absence of intact nuclei and the presence of nucleoli as dense refractive bodies. The sonicated sample was layered over a 0.88 M sucrose, 0.5 mM MgCl_2_ solution (S3 solution) and centrifuged for 10 minutes at 2,800 g at 4°C. The pellet contained the nucleoli while the supernatant consisted of the nucleoplasmic fraction. The nucleolar pellet was washed twice with S2 solution (5 minutes at 2,000 g) and protein extracts obtained by cell lysis buffer addition.

### Immunohistochemistry

Tumors were divided into half and either processed for protein lysates (as above) or fixed in formalin for staining using specific antibodies or H&E for histological evaluation. A paraffin-embedded tumor tissue array (BR8013, US Biomax) containing normal and tumor breast samples and MDA-MB-231 tumors were deparaffinized, rehydrated, processed for antigen retrieval, and peroxidase quenched as described [17]. Tissues were blocked (PBS with 1% BSA, and 0.1% Triton X-100) for 45 minutes at room temperature (RT) and incubated with anti-pY397 FAK (1:100), or anti-NS (1:100) in blocking buffer overnight. Biotinylated goat-anti-rabbit IgG, Vectastain ABC Elite, and diaminobenzidine were used to visualize antibody binding. Slides were counterstained with hematoxylin or methyl green. Images were captured using an upright microscope (Olympus BX43) with a color camera (Olympus SC100). For GFP analyses, cells were plated in coverslips, fixed with paraformaldehyde (PFA) (4%) for 10 minutes and cell nuclei were visualized by incubation with 1:50,000 dilution of Hoechst 33342 (Invitrogen). Images were sequentially captured at 20× magnification (UPLFL objective, 1.3 NA; Olympus) using a monochrome charge-coupled camera (ORCA ER; Hamamatsu), an inverted microscope (IX81; Olympus), and Slidebook software (v5.0, Intelligent Imaging). Images were pseudo-colored, overlaid, merged using Photoshop (Adobe), and quantified using Image J.

### Cell cycle analyses

Cells were collected as a single cell suspension by limited trypsin treatment and fixed in 70% ethanol and stored at −20°C overnight. Cells were incubated in 100 μl of PBS containing DNAse-free RNAse (100 μg/mL, Qiagen) and after 45 minutes, propidium iodide (PI, 10 μg/mL) was added prior to flow cytometry. Analyses were performed using FlowJo software (v9.5.1).

### Real-time quantitative PCR analyses

RNA was extracted from FAK-WT and FAK-KD MDA-MB-231 cells using the RNeasy Kit (Qiagen). NS and actin (used as normalization control) were amplified with specific primers (below). For PCR we used 480 SYBR green master mix (BioPioneer) with a LighterCycler 480 (Roche). Cycle conditions were 40 cycles of 94°C for 15 s, 56°C for 30 s, and 72°C for 30 s. The normalized mRNA level was defined as DCt = (target gene)-Ct (reference gene) and presented as fold difference between control and test samples (DCt control-DCt test). Actin forward 5′ GCGAGCACAGAGCCTCGCCTTTG 3′, actin reverse 5′ ACGACGAGCGCGGCGATATCAT 3′, NS forward 5′ TATCCATGGGGCTTACAAGG 3′ and NS reverse 5′ CTGGACTTCGCAGAGCAAG 3′.

### Database analyses

Expression array data were evaluated using the online Kaplan-Meier plotter [39]. The datasets (2014) were from Affymetrix HG-U133A, HG-U133 Plus 2.0, and HG-U133A 2.0 microarrays that share 22,277 probe sets in common. Overall survival (OS) information was from the Gene Expression Omnibus (GEO) (Affymetrix microarrays only), European Genome-Phenome Archive (EGA), and The Cancer Genome Atlas (TCGA) databases. The probe set used for PTK2 (FAK) analyses was 208820_at, NS was 217850_at, B23 was 221923_s_at and NCL (nucleolin) was 200610_s_at. Query parameters were: OS, split patients by median, auto-select best cutoff, and follow-up threshold of 10 years. For OS analyses, the signal range of the probes was as follows: PTK2 probe, 346–8042; NS probe, 1697–13661; B23 probe, 642–17106, and NCL probe, 1697–13661. The auto-cutoff value was 2515, 1802, 3772, and 6309 for PTK2, NS, B23, and NCL, respectively. Restriction analyses were estrogen receptor (ER) status (all), progesterone receptor (PR) status (all), lymph node status (all), grade (all), and intrinsic subtype (all). In total, 741 patient samples were analyzed.

### Statistics

Significant difference between groups was determined using one-way analysis of variance (ANOVA) with Tukey post hoc analysis. Differences between paired data were determined using the unpaired two-tailed Student’s *t*-test. All statistical analyses were performed using Prism (GraphPad Software, v5.0d). *P*-values <0.05 were considered significant.

## Results

### FAK inhibitor reduction in breast carcinoma anchorage-independent growth is associated with decreased nucleostemin levels

Nanomolar concentrations of pharmacological FAK inhibitors prevent 4T1 breast carcinoma growth under three-dimensional but not two-dimensional conditions [16]. This also occurs in mesothelioma [21] and ovarian carcinoma cells [17-19] expressing low levels of the merlin tumor suppressor protein [18,21]. The 50% inhibitory concentration (IC50) for FAK Y397 phosphorylation in MDA-MB-231 breast carcinoma cells is approximately 0.1 μM (Additional file 1: Figure S1A) and this is similar to results obtained with 4T1L murine breast carcinoma cells using a different (PND-1186, renamed VS-4718) FAK inhibitor [16].

**Figure 1:**
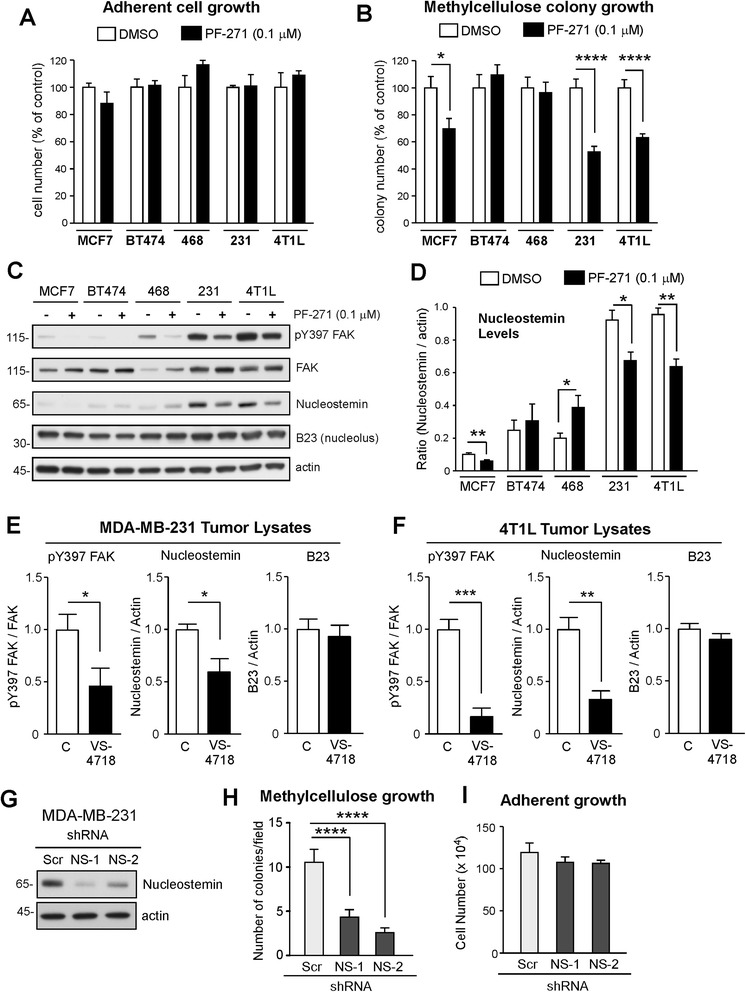
**Pharmacological focal adhesion kinase (FAK) inhibition selectively reduces nucleostemin (NS) levels and impacts anchorage-independent spheroid breast carcinoma growth. (A, B)** Growth of dimethyl sulfoxide (DMSO)- or PF-271 (0.1 μM)-treated cells: MCF7, BT474, 468 (MDA-MB-468), 231 (MDA-MB-231), and 4T1L in adherent conditions for 3 days **(A)** or methylcellulose for 25 days **(B)**. Values are means expressed as percent of DMSO control. **(C)** Representative lysates of the indicated cells treated with DMSO or PF-271 for 3 days and immunoblotted for pY397 FAK, total FAK, NS (light and dark exposures), B23, and actin. **(D)** Ratio of NS to actin levels in PF-271-treated and control cells determined by densitometry. **(E, F)** Immunoblots from tumor lysates (n = 5 independent controls and 5 treated with VS-4718) quantified by densitometry for MDA-MB-231 and 4T1L cells grown orthotopically in mice and treated with 5% sucrose (control) or 0.5 mg/kg VS-4718 [15]. Shown is the ratio of pY397 FAK to total FAK, NS to actin, and B23 to actin. The mean of vehicle-treated tumors was set to 1. **(G)** Anti-NS immunoblotting of MDA-MB-231 cells with lentiviral shRNA-mediated NS knockdown (NS-1 and NS-2) compared to scrambled (Scr) control. **(H, I)** Growth of MDA-MB-231 Scr, shRNA NS-1 or NS-2 cells in methylcellulose over 25 days **(H)** or adherent culture over 5 days **(I)**. For all graphs, values are means (+/− SEM, *P <0.05, **P <0.01, ***P <0.001, ****P <0.0001) of triplicate points from experiments repeated three times.

Here, we tested the effects of FAK inhibitor (0.1 μM, PF-271) addition on the two- and three-dimensional growth of human breast cancer cells. No growth differences were observed after 3 days in adherent two-dimensional culture (Figure 1A and Additional file 1: Figure S1B). This is consistent with studies showing that PF-271 (up to 1.0 μM) did not prevent prostate or pancreatic cancer cell growth in culture [37,40,41]. Although BT474 and MDA-MB-468 colony growth in methylcellulose was not affected, 0.1 μM PF-271 addition inhibited MCF7, MDA-MB-231, and 4T1L anchorage-independent three-dimensional colony growth (Figure 1B and Additional file 1: Figure S1C). Interestingly, NS knockdown also prevents three-dimensional growth of MCF7 and MDA-MB-231 cells [31]. Immunoblotting analyses revealed that whereas FAK Y397 phosphorylation was inhibited in all cells by 0.1 μM PF-271 treatment (Figure 1C), PF-271 reduced NS levels only in MCF7, MDA-MB-231, and 4T1L cells (Figure 1C and D). No PF-271-associated effects were observed on B23 (nucleophosmin) or nucleolin (Figure 1C and Additional file 1: Figure S1D) and low NS levels were not altered by PF-271 treatment of BT474 or MDA-MB-468 cells (Figure 1C and D). Together, these results support a connection between FAK activity and NS levels in a subset of breast carcinoma tumor cells.

Previous studies showed that orthotopic MDA-MB-231 and 4T1L tumor growth were inhibited by oral administration of the FAK inhibitor VS-4718 [15]. Additional analyses of tumor lysates from these experiments revealed decreased FAK Y397 phosphorylation, reduced NS levels, but no changes in B23 expression (Figure 1E and F). To confirm the importance of NS expression for MDA-MB-231 growth, two different shRNAs were stably-expressed by puromycin selection, and resulted in NS knockdown >75% in MDA-MB-231 cells (Figure 1G). NS knockdown but not expression of Scr shRNA control, prevented MDA-MB-231 colony growth in methylcellulose but not in two-dimensional adherent culture (Figure 1H and I). Interestingly, Kaplan-Meier analyses of a large tumor microarray database revealed that higher FAK, NS, and B23 mRNA levels were associated with decreased patient survival over 10 years (Additional file 2: Figure S2). As FAK inhibition can impact NS levels in tumors, our results support the notion that FAK and NS may be part of a signaling axis promoting breast carcinoma tumor progression.

### Genetic FAK inhibition decreases MDA-MB-231 growth in methylcellulose and reduces NS protein but not mRNA levels

**Figure 2:**
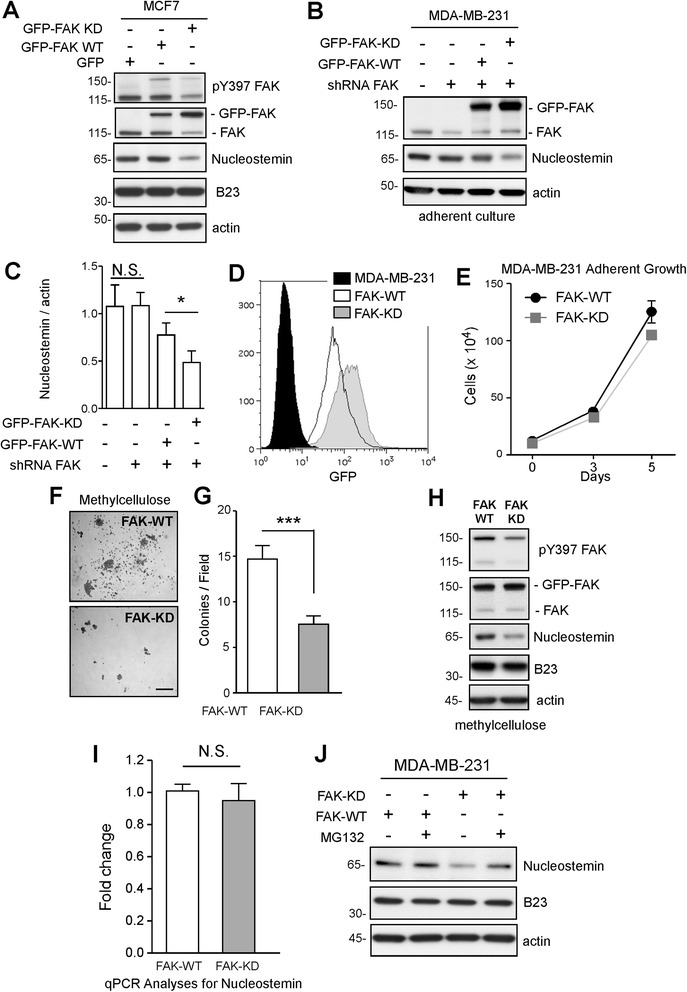
**Genetic focal adhesion kinase (FAK) inhibition impacts MDA-MB-231 spheroid growth and lowers nucleostemin (NS) protein levels. (A)** MCF7 cells over-expressing green fluorescent protein (GFP), GFP-FAK-wild-type (WT), or GFP-FAK-kinase-dead (KD) were analyzed for pY397 FAK, total FAK, NS, B23, and actin levels by immunoblotting. **(B)** MDA-MB-231 cells were transduced with lentiviral scrambled (Scr) or anti-human FAK shRNA. Murine GFP-FAK-WT (FAK-WT) or GFP-FAK-KD (FAK-KD) were stably re-expressed in FAK shRNA MDA-MB-231 cells. Immunoblotting of total FAK, NS, and actin. **(C)** Densitometry of NS to actin ratios. Values are means ± SD of triplicate points (*P <0.05) from three independent experiments. **(D)** Histogram of GFP fluorescence as determined by flow cytometry. **(E)** Adherent growth of FAK-WT and FAK-KD MDA-MB-231 cells. Values are means (± SEM) of triplicate points from two independent experiments. **(F)** Representative colony growth in methylcellulose (31 days) of FAK-WT and FAK-KD MDA-MB-231 cells. Scale is 0.5 mm. **(G)** Colony number of FAK-WT and FAK-KD MDA-MB-231 cells. Values are means ± SEM of triplicate points (***P <0.001) from two independent experiments. **(H)** Immunoblotting for pY397 FAK, total FAK, NS, B23, and actin in FAK-WT and FAK-KD MDA-MB-231 cells. **(I)** qPCR analyses of NS mRNA levels from FAK-WT and FAK-KD MDA-MB-231 cells. Fold change as compared to actin (± SEM) is shown. Data represent triplicate points from three independent experiments. N.S., no significant difference. **(J)** NS, B23, and actin immunoblotting analyses from FAK-WT and FAK-KD MDA-MB-231 cells cultured in methylcellulose for 3 days, and treated or not 3 hours prior to cell lysis (as indicated) with MG132 proteasome inhibitor (40 μM).

To support the linkage between FAK inhibition and decreased NS levels, lentiviral transduction was used to overexpress GFP or GFP fusions of FAK-WT or FAK-KD (K454R, kinase-dead) in MCF7 cells (Figure 2A). Immunoblotting revealed a selective reduction in NS but not B23 nucleolar protein levels by GFP-FAK-KD expression (Figure 2A). In a related approach, lentiviral anti-FAK shRNA was used to knock down endogenous FAK expression in MDA-MB-231 cells. Interestingly, stable FAK knockdown (approximately 50%) was not sufficient to alter steady-state NS levels (Figure 2B). However, upon stable re-expression of GFP-FAK-WT (herein termed FAK-WT) or GFP-FAK-KD (herein termed FAK-KD) in anti-FAK shRNA-expressing MDA-MB-231 cells, NS levels were reduced by approximately 60% in FAK-KD cells but not altered in FAK-WT cells (Figure 2B and C). These results support the notion that FAK activity facilitates the maintenance of NS expression in MCF7 and MDA-MB-231 cells.

By flow cytometry, GFP-FAK-KD was expressed at slightly higher levels than GFP-FAK-WT (Figure 2D). FAK-KD did not affect MDA-MB-231 adherent growth in culture (Figure 2E). However, FAK-KD significantly decreased anchorage-independent spheroid growth (*P* <0.001) (Figure 2F and G). Cell cycle analyses of FAK-WT and FAK-KD spheroids from methylcellulose colonies revealed a higher percentage of FAK-KD cells in G0/G1 and a lower percentage of cells in G2 compared to FAK-WT spheroids (Additional file 3: Figure S3). These results support the notion that FAK activity is selectively important in promoting anchorage-independent MDA-MB-231 cell proliferation.

Immunoblotting analyses of FAK-KD spheroids revealed decreased FAK pY397 phosphorylation and lower steady state levels of NS but not B23 compared to FAK-WT cells (Figure 2H). This difference was not associated with alterations in NS mRNA levels as analyzed by quantitative PCR (Figure 2I). However, addition of MG132 proteasome inhibitor to FAK-KD MDA-MB-231 cells for 3 hours was sufficient to restore NS levels equivalent to FAK-WT cells (Figure 2J). As it is known that NS is subject to ubiquitination and degradation [34], our results support the hypothesis that FAK activity is important in protecting NS protein from proteasomal degradation and that this linkage may enable MDA-MB-231 anchorage-independent spheroid growth.

### Genetic FAK inhibition impairs orthotopic tumor growth associated with lower tumor-associated NS

**Figure 3:**
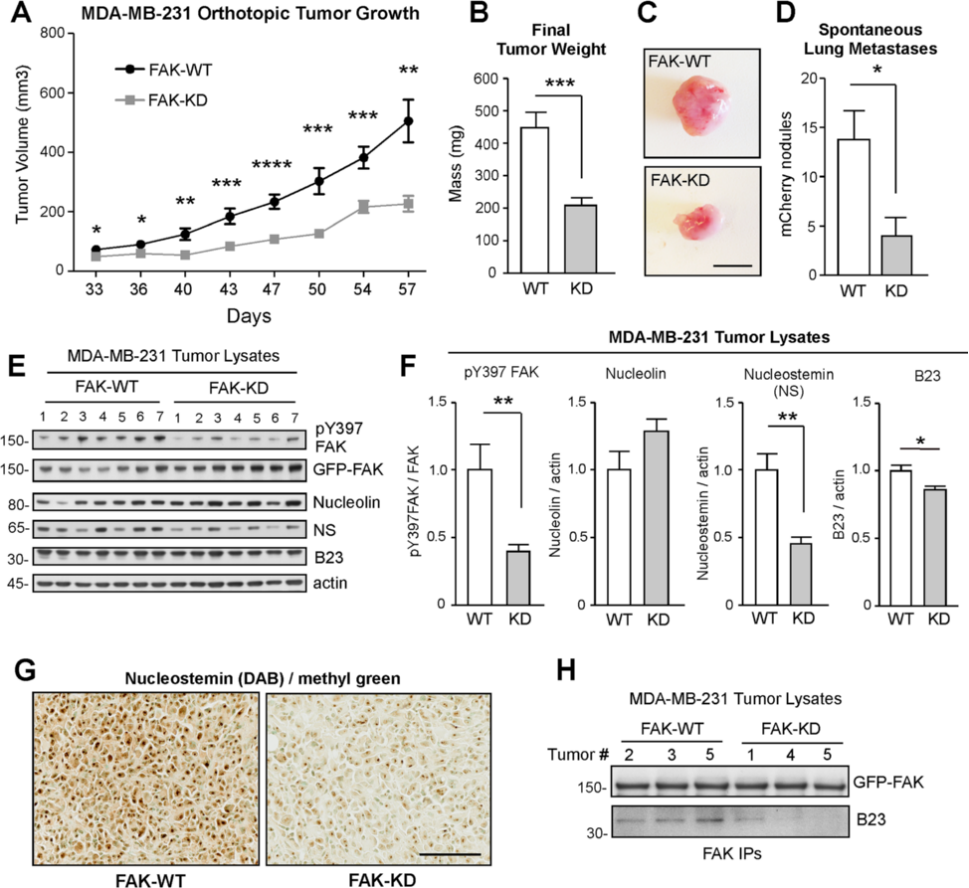
**Focal adhesion kinase (FAK)-kinase-dead (KD) MDA-MB-231 tumors are small with low nucleostemin (NS) levels. (A)** Mean volume of FAK-WT (black, n = 8) and FAK-KD (gray, n = 9) MDA-MB-231 cells grown as orthotopic breast tumors in NOD/SCID mice from day 33 to 57 (± SEM, *P <0.05, **P <0.01; ***P <0.001). **(B)** Final FAK-WT or FAK-KD tumor mass at day 57. Values are means (± SEM, ***P <0.001). **(C)** Representative images of FAK-WT and FAK-KD tumors at day 57 (scale 0.5 cm.) **(D)** Mean number of spontaneous breast to lung metastases as visualized by fluorescence (+/− SEM, * *P* <0.05). **(E)** Protein lysates from FAK-WT (n = 7) or FAK-KD (n = 7) MDA-MB-231 tumors analyzed by immunoblotting for pY397 FAK, total FAK, nucleolin, NS, B23, and actin. **(F)** Immunoblots in panel E were quantified by densitometry. Values (from seven tumors) are means (± SEM) and displayed as the ratio of FAK pY397 to total FAK, nucleolin to actin, NS to actin, and B23 to actin. FAK-WT values for each comparison were set to 1 (*P <0.05, **P <0.01). **(G)** Representative anti-NS immunohistochemical staining (diaminobenzidine, DAB) of FAK-WT and FAK-KD tumors. Tumors were counterstained with methyl green (scale 100 μm). **(H)** FAK immunoprecipitates (IPs) from three FAK-WT and three FAK-KD tumors were analyzed for associated B23 nucleolar protein by immunoblotting.

To determine whether MDA-MB-231 spheroid growth alterations results in altered tumor growth, FAK-WT or FAK-KD cells were orthotopically implanted into the breast fat pad of NOD/SCID mice (Figure 3). FAK-KD tumor volume was significantly lower compared to FAK-WT from Day 33 to Day 57 (Figure 3A). Mice bearing FAK-WT tumors at day 57 were larger (Figure 3B and C) and exhibited increased spontaneous metastasis from breast to lung (Figure 3D) compared to FAK-KD-bearing mice. Immunoblotting analyses of primary tumor lysates showed significantly decreased FAK Y397 phosphorylation and lower NS levels in seven independent FAK-KD tumors compared to FAK-WT samples (Figure 3E and F). Importantly, tumor levels of other nucleolar proteins such as B23 or nucleolin, respectively, were not different (Figure 3E and F). NS expression in FAK-WT tumors was higher than FAK-KD MDA-MB-231 tumors as confirmed by immunohistochemical staining of paraffin sections (Figure 3G). Analyses of FAK-IPs from FAK-WT and FAK-KD tumor lysates revealed the enhanced association of B23 with FAK-WT, but not with FAK-KD (Figure 3H). As B23 is a binding partner of NS in the nucleolus [42], we speculate that FAK interactions with B23, possibly in a sub-compartment such as the nucleolus, may facilitate NS regulation impacting breast tumor growth.

### Active FAK localization to the nucleolus in breast cancer cells

**Figure 4:**
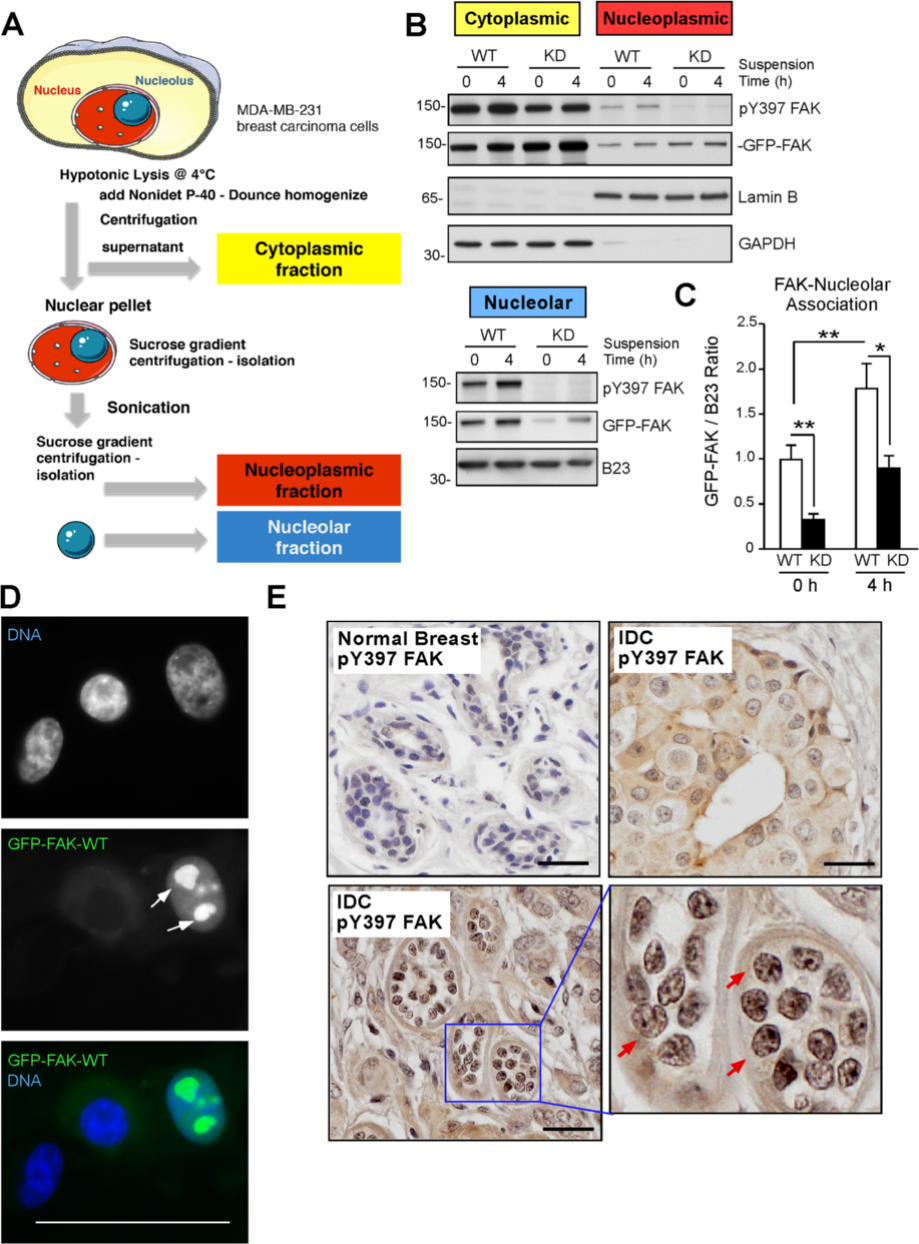
**Active focal adhesion kinase (FAK) associates with nucleoli in breast carcinoma cells. (A)** Cell fractionation protocol for cytoplasmic, nucleoplasmic, and nucleolar isolation. **(B)** FAK pY397, total FAK, Lamin B, glyceraldehyde-3-phosphate dehydrogenase (GAPDH), and B23 immunoblotting analyses of FAK-WT and FAK-KD reconstituted MDA-MB-231 fractionation from adherent (0 h) and cells held in suspension for 4 h. FAK-WT and FAK-KD were detected within cytoplasmic and nucleoplasmic fractions. Greater FAK-WT levels and Y397 phosphorylated FAK were associated with purified nucleoli compared to FAK-KD. **(C)** Ratio of GFP-FAK to B23 levels determined by densitometry: means ± SD from three independent experiments with FAK-WT at 0 h set to 1 (*P <0.05, **P <0.01). **(D)** Representative confocal microscopy of green fluorescent protein (GFP)-FAK wild-type (WT) fluorescence and nuclei staining. Merge shows Hoechst (blue) and nucleolar GFP-FAK-WT (green) localization (arrows). Medial confocal section (scale 50 μm). **(E)** Representative pY397 FAK staining in paraffin-embedded normal and human breast carcinoma tumors. Normal breast contains little pY397 FAK signal; invasive ductal carcinoma (IDC) exhibits both cytoplasmic (right) and nuclear-associated anti-pY397 FAK staining (brown). Inset, higher magnification shows punctate pY397 FAK staining (arrows) within nuclei. Slides were counterstained with hematoxylin (scale 25 μm).

In adherent cells, FAK localizes to sites of integrin adhesion. However, upon pharmacological FAK inhibition, conditions of cellular stress, or analyses of cells under anchorage-independent conditions, FAK nuclear distribution increases [22]. Nuclear FAK can alter gene expression and provide a kinase-independent survival signal to cells [23,24]. To determine the intracellular distribution of FAK in adherent and suspended MDA-MB-231 cells, biochemical fractionation was performed (Figure 4A). Cytoplasmic, nucleoplasmic (nuclear extract minus nucleolus), and purified nucleoli fractions were analyzed by immunoblotting for GFP-FAK, activated FAK (pY397 FAK phosphorylation), Lamin B (nucleoplasmic marker), GAPDH (cytoplasmic marker), and B23 (nucleolar marker) (Figure 4B). The majority of FAK-WT and FAK-KD were in the cytoplasmic fraction with lower levels detected in the nucleoplasmic fraction. Both FAK-WT and FAK-KD were present in the nucleoplasm and this did not change upon holding MDA-MB-231 cells in suspension for 4 h (Figure 4B). Interestingly, FAK-WT phosphorylated at Y397 was associated with purified nucleoli from adherent cells and the amount of nucleolar and Y397 phosphorylated FAK-WT increased upon holding MDA-MB-231 cells in suspension for 4 h (Figure 4B and C). Surprisingly, nucleoli from suspended cells contained only low levels of FAK-KD that was not phosphorylated (Figure 4B and C). These results support the hypothesis that FAK activity is important in promoting its nucleolar localization.

To determine if nucleolar FAK localization could be visualized in intact cells, adherent GFP-FAK-WT cells were imaged by confocal microscopy. Strong GFP-FAK-WT fluorescence was detected in a sub-compartment of the nucleus (Figure 4D, arrows and Additional file 4: Figure S4A). GFP-FAK-KD or expression of GFP alone in MDA-MB-231 cells did not exhibit this pattern of nucleolar localization as did GFP-FAK-WT (Additional file 4: Figure S4A and B). To determine if nucleoli-localized active FAK could be detected in breast tumors, a tissue micro-array containing normal breast and invasive ductal carcinoma (IDC) samples were stained with a monoclonal antibody to FAK pY397 (Figure 4E). Previous studies have shown that FAK expression is low in normal breast tissue and increases as a function of tumor grade and stage [7]. We find that normal breast tissue is weakly stained for pY397 FAK and the majority of IDC samples exhibit strong cytoplasmic or membrane-associated pY397 FAK staining (Figure 4E). However, in a subset of IDC tissues, strong nuclear- and nucleoli-localized pY397 FAK staining was detected (Figure 4E, arrows). These results support a signaling role for nucleolar-associated FAK in breast carcinoma cells that is distinct from a canonical integrin adhesion-dependent mechanism.

### Akt activation can prevent NS loss by FAK inhibition

**Figure 5:**
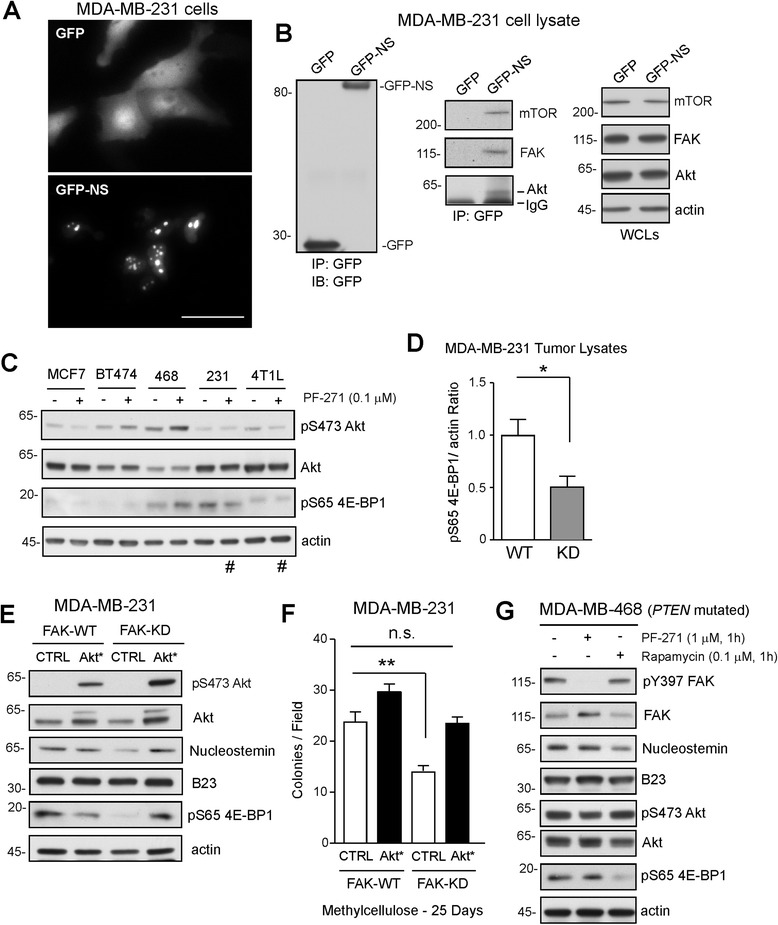
**Activated Akt can prevent nucleostemin (NS) loss by focal adhesion kinase (FAK) inhibition; connections to mammalian target of rapamycin (mTOR) as analyzed by pS65 4E-BP1 phosphorylation and rapamycin inhibition. (A)** Representative fluorescence confocal microscopy of MDA-MB-231 cells transfected with green fluorescent protein (GFP) or GFP-NS (24 h) (scale 50 μm). **(B)** Anti-GFP antibody co-immunoprecipitation (IP) of MDA-MB-231 cells transfected with GFP or GFP-NS. Shown is immunoblotting (IB) for GFP, mTOR, FAK, and Akt. **(C)** Lysates of dimethyl sulfoxide (DMSO)- or PF-271-treated (0.1 μM, 72 h) from adherent cells and immunoblotted for pS473 Akt, total Akt, pS65 4E-BP1, and actin. Reduction in pS473 Akt and pS65 4E-BP1 in PF-271-treated MDA-MB-231 and 4T1L cells (lanes indicated by #). **(D)** FAK-wild-type (WT) and FAK-kinase-dead (KD) MDA-MB-231 tumor lysates were immunoblotted for pS65 4E-BP1 and quantified by densitometry. Values are means (± SEM) and displayed as the ratio of pS65 4E-BP1 to actin. FAK-WT values were set to 1 (*P <0.05). **(E)** Stable expression of myristoylated - activated Akt (Akt*) in FAK-WT and FAK-KD MDA-MB-231 cells. Lysates were immunoblotted for pS473 Akt, total Akt, NS, B23, pS65 4E-BP1 and actin. **(F)** Colony formation of control (CTRL) and Akt*-expressing in FAK-WT and FAK-KD MDA-MB-231 cells. Values are means (± SEM, **P <0.01) of triplicate points. **(G)** MDA-MB-468 cells treated with PF-271 or rapamycin for 1 h (adherent) and lysates evaluated by pY397 FAK, total FAK, NS, B23, pS473 Akt, total Akt, pS65 4E-BP1, and actin immunoblotting. Blots are from one representative experiment repeated three times.

To determine if a complex between FAK and NS could be detected, MDA-MB-231 cells were transfected with GFP or GFP-NS (N-terminal fusion). GFP-NS exhibited sub-compartment localization within nuclei, whereas GFP was distributed throughout cells (Figure 5A). Co-immunoprecipitation assays with antibodies to GFP revealed a complex of mTOR, FAK, and Akt with GFP-NS and not GFP alone (Figure 5B). To determine if FAK inhibition alters Akt or mTOR activation in breast carcinoma cells, lysates of MCF7, BT474, MDA-MB-468, MDA-MB-231, and 4T1L treated with DMSO (control) or 0.1 μM PF-271 FAK inhibitor for 3 days were evaluated by immunoblotting (Figure 5C). BT474 and MDA-MB-468 cells maintained elevated phosphorylated serine-473 (pS473) Akt in the presence of PF-271 (Figure 5C). This is consistent with *PIK3CA* and *PTEN* mutations in BT474 and MDA-MB-468 cells, respectively (Table 1). However, reduced pS473 Akt phosphorylation occurred in MCF7, MDA-MB-231, and 4T1L cells with PF-271 addition (Figure 5C). In MDA-MB-231 and 4T1L cells, 0.1 μM PF-271 also decreased serine-65 (pS65) phosphorylation of 4E-binding protein 1 (4E-BP1) [43], a translation repressor protein and target the mTOR complex (Figure 5C, #). In MDA-MB-468 cells, PF-271 did not reduce either Akt or 4E-BP1 phosphorylation (Figure 5C) and these cells were insensitive to PF-271-mediated regulation of NS (Figure 1C). In MDA-MB-231 tumor lysates, genetic FAK inhibition (FAK-KD) was associated with decreased pS65 4E-BP1 phosphorylation (Figure 5D). These results support the notion that FAK may stimulate Akt-mTOR pathway activation that may act to stabilize NS protein levels.

To test the importance of Akt activity in this linkage, constitutively-active Akt (myristylated-Akt, Akt*) was stably expressed in FAK-WT and FAK-KD MDA-MB-231 cells. Enhanced Akt activity was confirmed by elevated pS473 Akt phosphorylation in both FAK-WT and FAK-KD cells (Figure 5E). Importantly, Akt* expression resulted in elevated steady-state NS levels and increased 4E-BP1 phosphorylation in FAK-KD cells. Akt* expression was sufficient to rescue FAK-KD MDA-MB-231 colony growth in methylcellulose equal to control FAK-WT levels (Figure 5F). These results demonstrate that activated Akt expression can promote colony growth and prevent NS loss associated with FAK inhibition.

### Rapamycin lowers NS levels in MDA-MB-468 cells with activated Akt

PF-271-mediated FAK inhibition did not alter NS levels in MDA-MB-468 cells that exhibit high levels of Akt and 4E-BP1 phosphorylation. DNA sequencing of MDA-MB-468 cells has revealed mutations in the phosphatase and tensin homolog (PTEN) protein that acts as a negative regulator of the Akt signaling pathway (Table 1). To evaluate the importance of mTOR activation in maintaining NS protein levels, MDA-MB-468 cells were treated with rapamycin (0.1 μM for 1 h), an inhibitor of mTOR complex (Figure 5G). Rapamycin treatment decreased NS levels and reduced 4E-BP1 phosphorylation. This was independent of effects on FAK or Akt phosphorylation. These results support the importance of mTOR activity in maintaining NS levels in MDA-MB-468 cells.

## Discussion

FAK inhibitors are being evaluated in several clinical trials with an underlying rationale that effects are mediated through alterations in both tumor and stromal cell function [6]. FAK inhibitory effects on tumor cells involve the prevention of cell motility and invasion *in vitro* as well as tumor growth and metastasis in mouse models. FAK inhibitor administration can slow tumor growth and trigger increased tumor cell apoptosis *in vivo* [15,37]. Although this may be due in part to angiogenesis prevention [44], studies are beginning to uncover selective roles for FAK signaling when tumor cells are grown as spheroids *in vitro* [16-19]. Importantly, nanomolar sensitivity to FAK inhibition is not observed using standard monolayer cell culture. However, some tumor cells exhibit insensitivity as three-dimensional spheroids to micromolar FAK inhibitor levels [18]. Elucidating the molecular basis for these differential effects will provide fundamental knowledge of how best to apply FAK inhibitor treatment in clinical settings.

Herein, we identify a new linkage between FAK activity and the nucleolar protein, nucleostemin (NS), in the regulation of anchorage-independent spheroid and orthotopic breast carcinoma growth as tumors. Genetic or pharmacological FAK inhibition was associated with decreased NS levels but not other nucleolar proteins such as B23 (nucleophosmin) or nucleolin in cell culture and in tumors. NS knockdown prevented MDA-MB-231 cell growth as spheroids but was not essential for growth in two-dimensional culture. FAK inhibition did not alter NS mRNA levels and proteasomal inhibition stabilized NS upon FAK inhibition. These results support the importance of FAK activity in protecting NS protein from proteasomal degradation and we hypothesize that this may enable MDA-MB-231 anchorage-independent growth. However, NS over-expression in MDA-MB-231 cells did not promote anchorage-independent growth in the presence of FAK inhibition (I. Tancioni, unpublished) and cells expressing high basal NS levels (MDA-MB-231 and 4T1L) are not resistant to FAK inhibitor growth inhibition. Thus, high NS levels are not predictive of FAK inhibitor resistance.

Nevertheless, it is intriguing that both FAK and NS can control common stem cell-like properties of tumor cells such as anchorage-independent growth [21,31]. Since tumor cells are not making canonical focal adhesions as three-dimensional spheroids, we investigated the distribution of active FAK by cellular fractionation. An increased percentage of active GFP-FAK-WT was associated with purified nucleoli following cell suspension for 4 hours. Surprisingly, although GFP-FAK-KD was present in the nucleoplasmic fraction, only low levels of GFP-FAK-KD were found in purified nucleoli. The mechanism(s) directing active FAK to the nucleolus remain under investigation. For FAK-WT, nucleolar localization was detected by confocal microscopy and a subset of invasive ductal breast carcinoma tumors exhibited nucleolar staining with antibodies to pY397 FAK. These results are consistent with phosphoproteomic studies showing that Y397 FAK was elevated in stem cell spheroid clusters [45] and that nuclear active FAK is associated with a poor prognosis in human colorectal cancer [46]. As FAK-WT co-immunoprecipitates with B23, and NS forms a complex with B23 in the nucleolus [42], our studies support a signaling role for active nucleolar-associated FAK that is distinct from a canonical integrin adhesion linkage mechanism.

Expression of a GFP-tagged NS construct localized to nucleolar sites in MDA-MB-231 cells and formed a complex with FAK, Akt, and mTOR as determined by co-immunoprecipitation. Interestingly, mTOR Complex 1 can localize to the nucleolus and nuclear Akt interacts with B23, leading to protection from proteolytic cleavage and enhancement of cell survival [47,48]. This led us to explore whether connections may exist between FAK, Akt, and mTOR in the regulation of NS levels. Indeed, expression of constitutively-active Akt promoted colony growth and stabilized NS levels in the presence of inhibited FAK. Further, rapamycin inhibition of mTOR lowered NS levels in *PTEN*-mutated MDA-MB-468 breast carcinoma cells. These results support a FAK to Akt to mTOR signaling linkage in the regulation of NS levels. As elevated FAK expression in breast cancer is associated with a triple-negative cell phenotype [49], our results support a model whereby FAK activity-mediated modulation of NS levels may be part of a mesenchymal- or stem cell-associated regulatory circuit. Combinational use of FAK and PI3K-mTOR inhibitors [50] may have additive effects in breast cancers that show resistance to standard therapies.

## Conclusions

This study shows that FAK activity regulates nucleostemin (NS) levels, a nucleolar protein involved in promoting breast tumor growth. FAK activity and NS are selectively required for anchorage-independent MDA-MB-231 spheroid growth and cell fractionation analyses revealed active FAK association with nucleoli upon holding MDA-MB-231 cells in suspension. Activation of Akt stabilized NS levels and promoted anchorage-independent growth in the presence of inactivated FAK. As rapamycin lowered NS levels in *PTEN*-mutated MDA-MB-468 breast carcinoma cells, our studies support a role for nucleolar-associated FAK signaling and Akt-mTOR activation in the maintenance of NS protein expression. Reduction in NS levels may serve as a protein biomarker of FAK inhibitor effectiveness in breast cancer therapy.

## Additional files

Additional file 1: Figure S1. Pharmacological focal adhesion kinase (FAK) inhibition prevents breast carcinoma growth in methylcellulose but not in adherent conditions. (**A**) Dose-dependent inhibition of FAK pY397 phosphorylation by PF-271. MDA-MB-231 cells were treated with vehicle (dimethyl sulfoxide (DMSO)) or increasing concentrations of PF-271 for 1 hour and lysates immunoblotted for pY397 FAK, total FAK, and actin. The ratio between pY397FAK and FAK of DMSO was set to 1. (**B**) The indicated breast carcinoma cells were grown in presence of DMSO or PF-271 (0.1 μM) for 3 days and imaged by phase contrast microscopy. Scale is 100 μm. (**C**) The indicated breast carcinoma cells were grown in presence of DMSO or PF-271 (0.1 μM) for 25 days in methylcellulose and imaged by phase contrast microscopy. Scale is 500 μm. (**D**) Lysates of DMSO- or 0.1 μM PF-271-treated (3 days) MCF7, BT474, 468 (MDA-MB-468), 231 (MDA-MB-231) cells and immunoblotted for pY397 FAK, total FAK, nucleolin, and actin.
Additional file 2: Figure S2. Association of focal adhesion kinase (FAK), nucleostemin, and B23 mRNA levels with breast cancer patient overall survival. Kaplan-Meier analyses of FAK, nucleostemin, B23, and nucleolin mRNA levels in 741 patient samples. High (red) versus low (black) mRNA expression shows overall patient survival over 120 months. Log-rank *P*-values for significance are shown (inset).
Additional file 3: Figure S3. Focal adhesion kinase- (FAK)-kinase-dead (KD) MDA-MB-231 cells show higher percentage of cells in G0/G1 phase than wild-type (WT)-FAK cells. FAK-WT and FAK-KD MDA-MB-231 cells cultured in methylcellulose for 3 days were collected, stained with propidium iodide, and analyzed by flow cytometry. Representative histogram of cell cycle analyses and percentage of cells in Sub-G1, G0/G1, S, and G2 phases of the cell cycle as analyzed by FlowJo software.
Additional file 4: Figure S4. Green fluorescent protein-focal adhesion kinase-wildtype (GFP-FAK-WT) nucleolar localization. (**A**) Representative confocal microscopy images of re-expressing GFP-FAK-WT and GFP-FAK-kinase-dead (KD) MDA-MB-231 cells (Hoechst nuclear stain, blue) reveal nucleolar GFP-FAK-WT localization (arrows). GFP-FAK-KD is primarily cytoplasmic in the confocal image. (**B**) Representative confocal images of MDA-MB-231 expressing GFP-FAK-WT or GFP. Scale is 50 μm.

## Abbreviations

4E-BP1: 4E-binding protein 1
Akt: Also known as protein kinase B
ANOVA: analysis of variance
DMEM: Dulbecco's modified Eagle's medium
DMSO: dimethyl sulfoxide
ER: estrogen receptor
FACS: fluorescence-activated cell sorting
FAK: focal adhesion kinase
FBS: fetal bovine serum
GFP: green fluorescent protein
GTP: guanine triphosphate
H&E: hematoxylin and eosin
HEPES: 4-(2-hydroxyethyl)-1-piperazineethanesulfonic acid
HER2: human epidermal growth factor receptor 2
IC50: 50% inhibitory concentration
IDC: invasive ductal carcinoma
IgG: immunoglobulin G
KD: kinase-dead
MDM2: mouse double minute 2
mTOR: mammalian target of rapamycin
NS: nucleostemin
OS: overall survival
PBS: phosphate-buffered saline
PI: propidium iodide
PI3K: phosphatidylinositol-3-Kinase
*PIK3CA*: Phosphoinositide-3-kinase-catalytic-alpha
poly-HEMA: poly-hyodroxyethyl methacrylic acid
PR: progesterone receptor
PTEN: phosphatase and tensin homolog
Scr: scrambled
SEM: standard error of the mean
shRNA: short-hairpin RNA
TNBC: triple-negative breast cancer
TP53: tumor protein 53
WT: wild-type

## References

[1] Siegel R, Ma J, Zou Z, Jemal A (2014). Cancer statistics, 2014. CA Cancer J Clin.

[2] Vargo-Gogola T, Rosen JM (2007). Modelling breast cancer: one size does not fit all. Nat Rev Cancer.

[3] Dent R, Trudeau M, Pritchard KI, Hanna WM, Kahn HK, Sawka CA (2007). Triple-negative breast cancer: clinical features and patterns of recurrence. Clin Cancer Res.

[4] Weigelt B, Peterse JL, van ‘t Veer LJ (2005). Breast cancer metastasis: markers and models. Nat Rev Cancer.

[5] Desgrosellier JS, Cheresh DA (2010). Integrins in cancer: biological implications and therapeutic opportunities. Nat Rev Cancer.

[6] Sulzmaier FJ, Jean C, Schlaepfer DD (2014). FAK in cancer: mechanistic findings and clinical applications. Nat Rev Cancer.

[7] Lark AL, Livasy CA, Dressler L, Moore DT, Millikan RC, Geradts J (2005). High focal adhesion kinase expression in invasive breast carcinomas is associated with an aggressive phenotype. Mod Pathol.

[8] Yom CK, Noh DY, Kim WH, Kim HS (2011). Clinical significance of high focal adhesion kinase gene copy number and overexpression in invasive breast cancer. Breast Cancer Res Treat.

[9] Cance WG, Harris JE, Iacocca MV, Roche E, Yang X, Chang J (2000). Immunohistochemical analyses of focal adhesion kinase expression in benign and malignant human breast and colon tissues: correlation with preinvasive and invasive phenotypes. Clin Cancer Res.

[10] Theocharis SE, Klijanienko JT, Padoy E, Athanassiou S, Sastre-Garau XX (2009). Focal adhesion kinase (FAK) immunocytochemical expression in breast ductal invasive carcinoma (DIC): correlation with clinicopathological parameters and tumor proliferative capacity. Med Sci Monit.

[11] Provenzano PP, Inman DR, Eliceiri KW, Beggs HE, Keely PJ (2008). Mammary epithelial-specific disruption of focal adhesion kinase retards tumor formation and metastasis in a transgenic mouse model of human breast cancer. Am J Pathol.

[12] Lahlou H, Sanguin-Gendreau V, Zuo D, Cardiff RD, McLean GW, Frame MC (2007). Mammary epithelial-specific disruption of the focal adhesion kinase blocks mammary tumor progression. Proc Natl Acad Sci U S A.

[13] Luo M, Fan H, Nagy T, Wei H, Wang C, Liu S (2009). Mammary epithelial-specific ablation of the focal adhesion kinase suppresses mammary tumorigenesis by affecting mammary cancer stem/progenitor cells. Cancer Res.

[14] Pylayeva Y, Gillen KM, Gerald W, Beggs HE, Reichardt LF, Giancotti FG (2009). Ras- and PI3K-dependent breast tumorigenesis in mice and humans requires focal adhesion kinase signaling. J Clin Invest.

[15] Walsh C, Tanjoni I, Uryu S, Tomar A, Nam JO, Luo H (2010). Oral delivery of PND-1186 FAK inhibitor decreases tumor growth and spontaneous breast to lung metastasis in pre-clinical models. Cancer Biol Ther.

[16] Tanjoni I, Walsh C, Uryu S, Tomar A, Nam JO, Mielgo A (2010). PND-1186 FAK inhibitor selectively promotes tumor cell apoptosis in three-dimensional environments. Cancer Biol Ther.

[17] Ward KK, Tancioni I, Lawson C, Miller NL, Jean C, Chen XL (2013). Inhibition of focal adhesion kinase (FAK) activity prevents anchorage-independent ovarian carcinoma cell growth and tumor progression. Clin Exp Metastasis.

[18] Shah NR, Tancioni I, Ward KK, Lawson C, Chen XL, Jean C (2014). Analyses of merlin/NF2 connection to FAK inhibitor responsiveness in serous ovarian cancer. Gynecol Oncol.

[19] Tancioni I, Uryu S, Sulzmaier FJ, Shah NR, Lawson C, Miller NL (2014). FAK Inhibition Disrupts a beta5 Integrin Signaling Axis Controlling Anchorage-Independent Ovarian Carcinoma Growth. Mol Cancer Ther.

[20] Wendt MK, Smith JA, Schiemann WP (2010). Transforming growth factor-beta-induced epithelial-mesenchymal transition facilitates epidermal growth factor-dependent breast cancer progression. Oncogene.

[21] Shapiro IM, Kolev VN, Vidal CM, Kadariya Y, Ring JE, Wright Q, et al. Merlin deficiency predicts for FAK inhibitor sensitivity: a synthetic lethal relationship. Sci Trans Med. 2014;6:237ra68. 10.1126/scitranslmed.3008639 PMC4165339 24848258

[22] Lim ST, Chen XL, Lim Y, Hanson DA, Vo TT, Howerton K (2008). Nuclear FAK promotes cell proliferation and survival through FERM-enhanced p53 degradation. Mol Cell.

[23] Luo SW, Zhang C, Zhang B, Kim CH, Qiu YZ, Du QS (2009). Regulation of heterochromatin remodelling and myogenin expression during muscle differentiation by FAK interaction with MBD2. EMBO J.

[24] Lim ST, Miller NL, Chen XL, Tancioni I, Walsh CT, Lawson C (2012). Nuclear-localized focal adhesion kinase regulates inflammatory VCAM-1 expression. J Cell Biol.

[25] Visvader JE, Lindeman GJ (2008). Cancer stem cells in solid tumours: accumulating evidence and unresolved questions. Nat Rev Cancer.

[26] Boisvert FM, van Koningsbruggen S, Navascues J, Lamond AI (2007). The multifunctional nucleolus. Nat Rev Mol Cell Biol.

[27] Hein N, Hannan KM, George AJ, Sanij E, Hannan RD (2013). The nucleolus: an emerging target for cancer therapy. Trends Mol Med.

[28] Tsai RY, McKay RD (2002). A nucleolar mechanism controlling cell proliferation in stem cells and cancer cells. Genes Dev.

[29] Tsai RY (2014). Turning a new page on nucleostemin and self-renewal. J Cell Sci.

[30] Okamoto N, Yasukawa M, Nguyen C, Kasim V, Maida Y, Possemato R (2011). Maintenance of tumor initiating cells of defined genetic composition by nucleostemin. Proc Natl Acad Sci U S A.

[31] Lin T, Meng L, Li Y, Tsai RY (2010). Tumor-initiating function of nucleostemin-enriched mammary tumor cells. Cancer Res.

[32] Kobayashi T, Masutomi K, Tamura K, Moriya T, Yamasaki T, Fujiwara Y (2014). Nucleostemin expression in invasive breast cancer. BMC Cancer.

[33] Tsai RY, McKay RD (2005). A multistep, GTP-driven mechanism controlling the dynamic cycling of nucleostemin. J Cell Biol.

[34] Huang M, Itahana K, Zhang Y, Mitchell BS (2009). Depletion of guanine nucleotides leads to the Mdm2-dependent proteasomal degradation of nucleostemin. Cancer Res.

[35] Lo D, Dai MS, Sun XX, Zeng SX, Lu H (2012). Ubiquitin- and MDM2 E3 ligase-independent proteasomal turnover of nucleostemin in response to GTP depletion. J Biol Chem.

[36] Huang M, Whang P, Chodaparambil JV, Pollyea DA, Kusler B, Xu L (2011). Reactive oxygen species regulate nucleostemin oligomerization and protein degradation. J Biol Chem.

[37] Roberts WG, Ung E, Whalen P, Cooper B, Hulford C, Autry C (2008). Antitumor activity and pharmacology of a selective focal adhesion kinase inhibitor, PF-562,271. Cancer Res.

[38] Andersen JS, Lyon CE, Fox AH, Leung AK, Lam YW, Steen H (2002). Directed proteomic analysis of the human nucleolus. Curr Biol.

[39] Gyorffy B, Lanczky A, Szallasi Z (2012). Implementing an online tool for genome-wide validation of survival-associated biomarkers in ovarian-cancer using microarray data from 1287 patients. Endocr Relat Cancer.

[40] Stokes JB, Adair SJ, Slack-Davis JK, Walters DM, Tilghman RW, Hershey ED (2011). Inhibition of focal adhesion kinase by PF-562,271 inhibits the growth and metastasis of pancreatic cancer concomitant with altering the tumor microenvironment. Mol Cancer Ther.

[41] Slack-Davis JK, Martin KH, Tilghman RW, Iwanicki M, Ung EJ, Autry C (2007). Cellular characterization of a novel focal adhesion kinase inhibitor. J Biol Chem.

[42] Ma H, Pederson T (2008). Nucleophosmin is a binding partner of nucleostemin in human osteosarcoma cells. Mol Biol Cell.

[43] Hay N, Sonenberg N (2004). Upstream and downstream of mTOR. Genes Dev.

[44] Cabrita MA, Jones LM, Quizi JL, Sabourin LA, McKay BC, Addison CL (2011). Focal adhesion kinase inhibitors are potent anti-angiogenic agents. Mol Oncol.

[45] Beck HC, Gosau M, Kristensen LP, Morsczeck C (2014). A site-specific phosphorylation of the focal adhesion kinase controls the formation of spheroid cell clusters. Neurochem Res.

[46] Albasri A, Fadhil W, Scholefield JH, Durrant LG, Ilyas M (2014). Nuclear expression of phosphorylated focal adhesion kinase is associated with poor prognosis in human colorectal cancer. Anticancer Res.

[47] Lee SB, Xuan Nguyen TL, Choi JW, Lee KH, Cho SW, Liu Z (2008). Nuclear Akt interacts with B23/NPM and protects it from proteolytic cleavage, enhancing cell survival. Proc Natl Acad Sci U S A.

[48] Iadevaia V, Zhang Z, Jan E, Proud CG (2012). mTOR signaling regulates the processing of pre-rRNA in human cells. Nucleic Acids Res.

[49] Golubovskaya VM, Ylagan L, Miller A, Hughes M, Wilson J, Wang D (2014). High focal adhesion kinase expression in breast carcinoma is associated with lymphovascular invasion and triple-negative phenotype. BMC Cancer.

[50] Miller TW, Rexer BN, Garrett JT, Arteaga CL (2011). Mutations in the phosphatidylinositol 3-kinase pathway: role in tumor progression and therapeutic implications in breast cancer. Breast Cancer Res.

[51] Broad-Novartis Cancer Cell Line Encyclopedia. http://www.broadinstitute.org/ccle.

[52] Neve RM, Chin K, Fridlyand J, Yeh J, Baehner FL, Fevr T (2006). A collection of breast cancer cell lines for the study of functionally distinct cancer subtypes. Cancer Cell.

