# Supplementary figures and images for: FAK activity protects nucleostemin in facilitating breast cancer spheroid and tumor growth
Source: Breast Cancer Res. 2015 Mar 28;17:47. doi: 10.1186/s13058-015-0551-x (PMC4407832; doi:10.1186/s13058-015-0551-x)

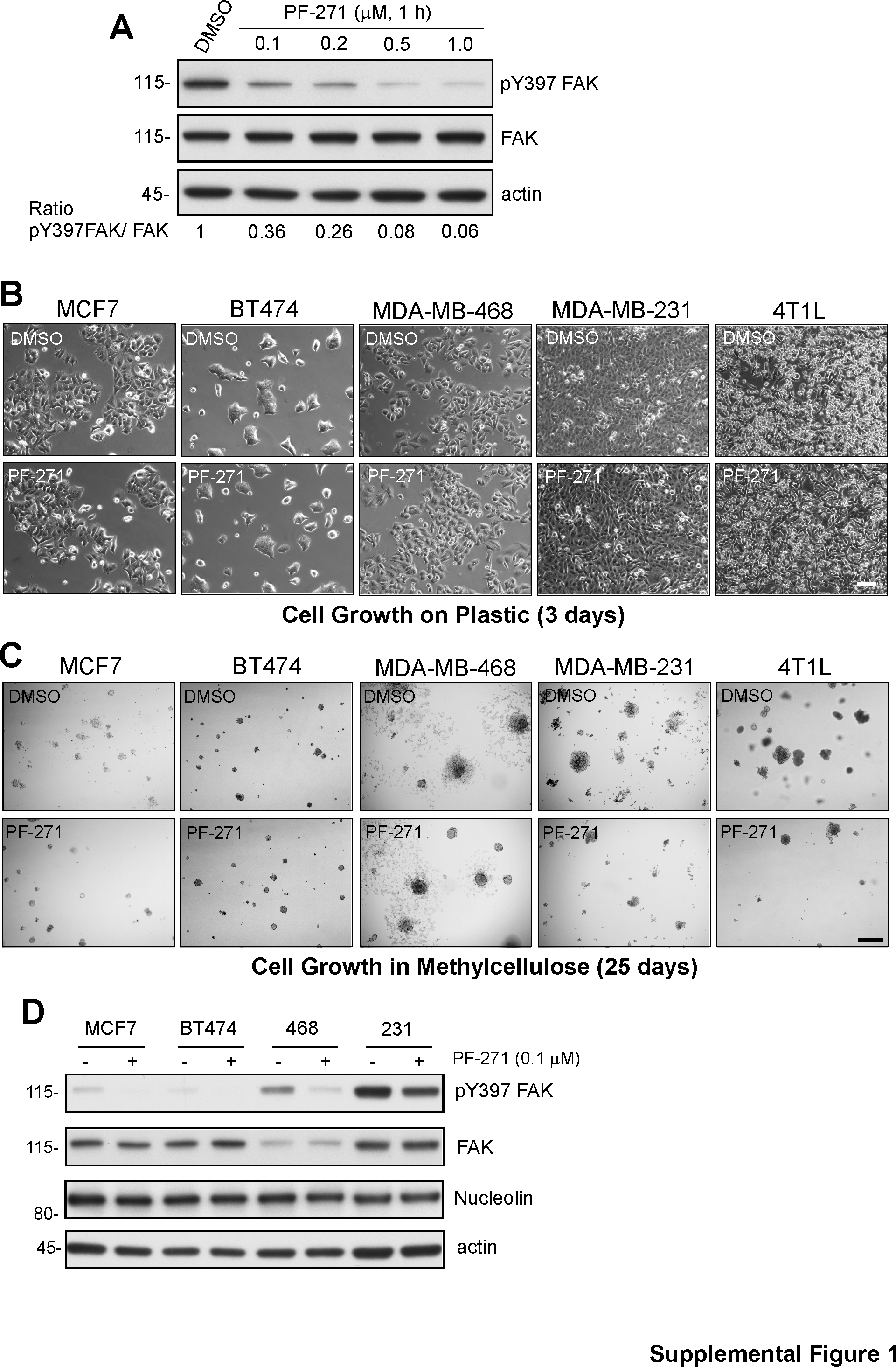

Supplement: Additional file 1: Figure S1. — Pharmacological focal adhesion kinase (FAK) inhibition prevents breast carcinoma growth in methylcellulose but not in adherent conditions. (A) Dose-dependent inhibition of FAK pY397 phosphorylation by PF-271. MDA-MB-231 cells were treated with vehicle (dimethyl sulfoxide (DMSO)) or increasing concentrations of PF-271 for 1 hour and lysates immunoblotted for pY397 FAK, total FAK, and actin. The ratio between pY397FAK and FAK of DMSO was set to 1. (B) The indicated breast carcinoma cells were grown in presence of DMSO or PF-271 (0.1 μM) for 3 days and imaged by phase contrast microscopy. Scale is 100 μm. (C) The indicated breast carcinoma cells were grown in presence of DMSO or PF-271 (0.1 μM) for 25 days in methylcellulose and imaged by phase contrast microscopy. Scale is 500 μm. (D) Lysates of DMSO- or 0.1 μM PF-271-treated (3 days) MCF7, BT474, 468 (MDA-MB-468), 231 (MDA-MB-231) cells and immunoblotted for pY397 FAK, total FAK, nucleolin, and actin. [file 13058_2015_551_MOESM1_ESM.tiff]

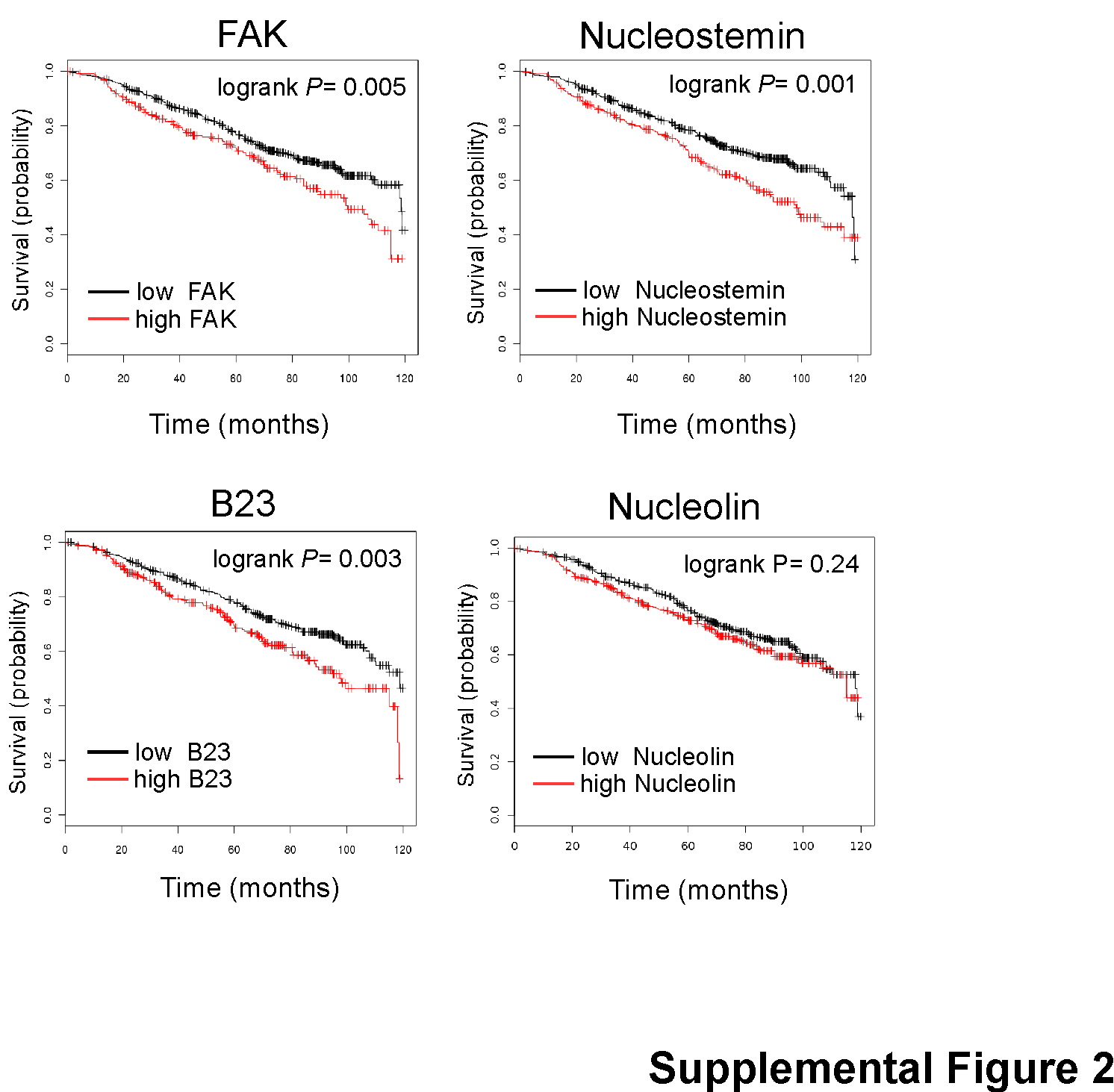

Supplement: Additional file 2: Figure S2. — Association of focal adhesion kinase (FAK), nucleostemin, and B23 mRNA levels with breast cancer patient overall survival. Kaplan-Meier analyses of FAK, nucleostemin, B23, and nucleolin mRNA levels in 741 patient samples. High (red) versus low (black) mRNA expression shows overall patient survival over 120 months. Log-rank P-values for significance are shown (inset). [file 13058_2015_551_MOESM2_ESM.tiff]

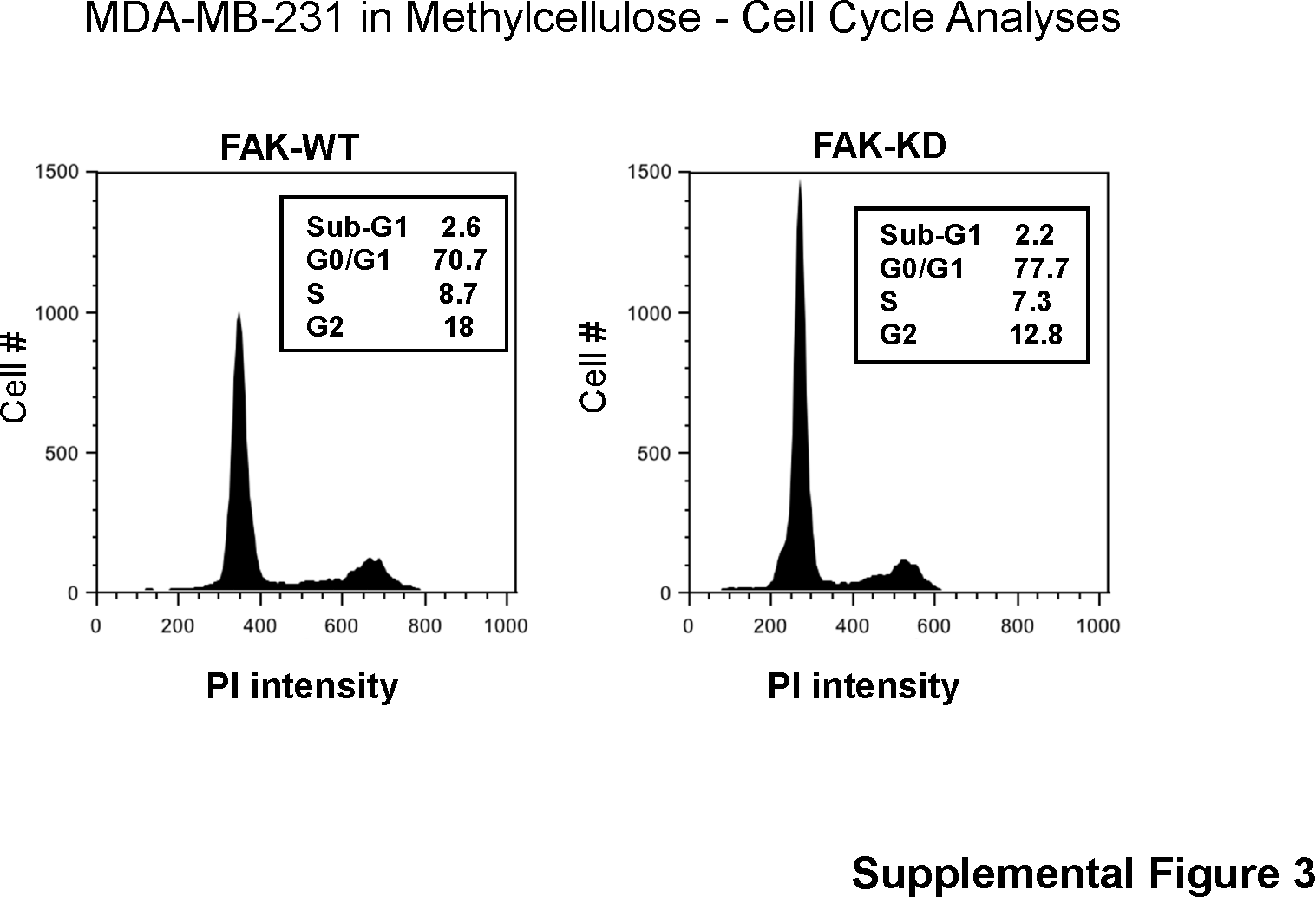

Supplement: Additional file 3: Figure S3. — Focal adhesion kinase- (FAK)-kinase-dead (KD) MDA-MB-231 cells show higher percentage of cells in G0/G1 phase than wild-type (WT)-FAK cells. FAK-WT and FAK-KD MDA-MB-231 cells cultured in methylcellulose for 3 days were collected, stained with propidium iodide, and analyzed by flow cytometry. Representative histogram of cell cycle analyses and percentage of cells in Sub-G1, G0/G1, S, and G2 phases of the cell cycle as analyzed by FlowJo software. [file 13058_2015_551_MOESM3_ESM.tiff]

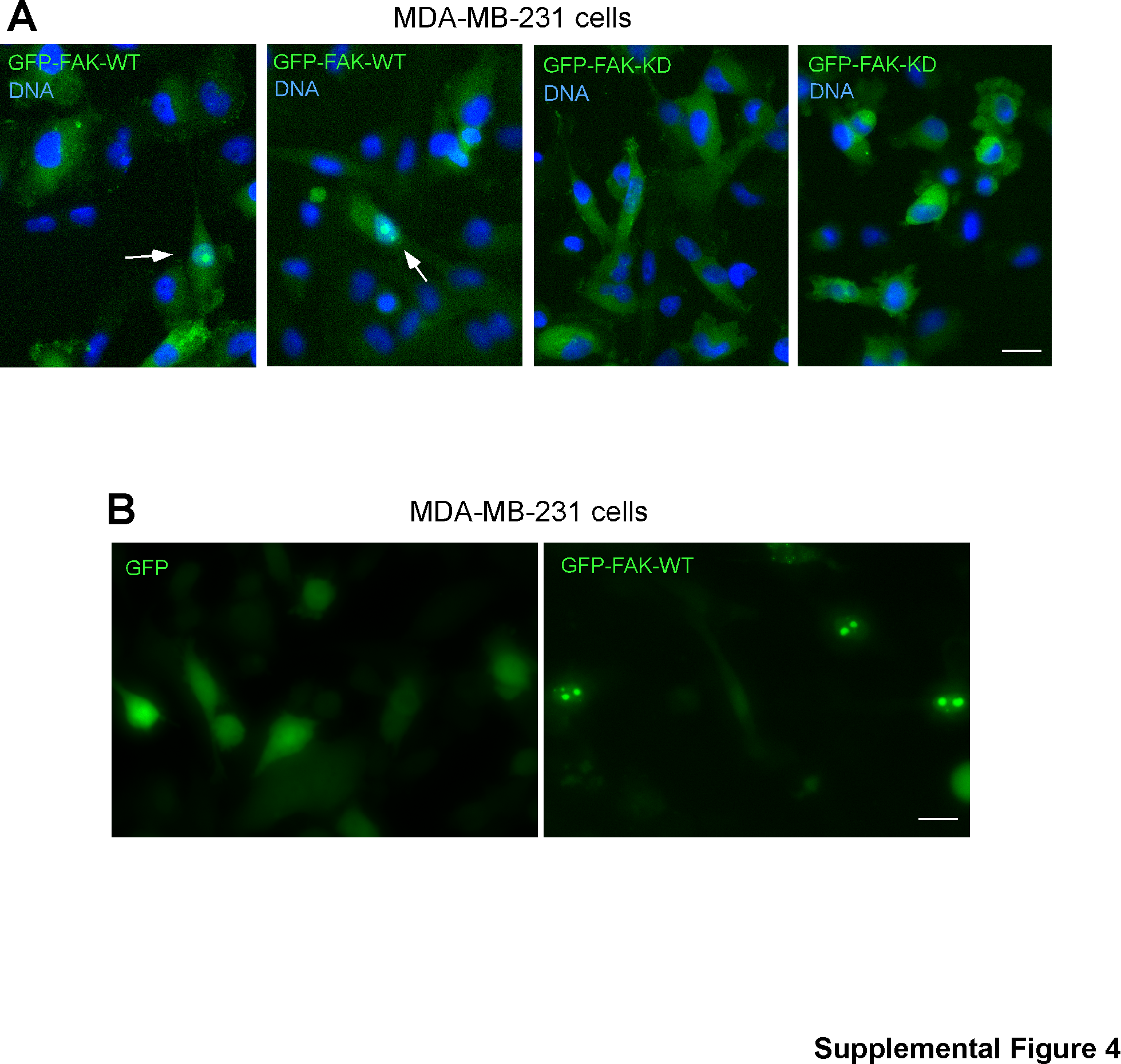

Supplement: Additional file 4: Figure S4. — Green fluorescent protein-focal adhesion kinase-wildtype (GFP-FAK-WT) nucleolar localization. (A) Representative confocal microscopy images of re-expressing GFP-FAK-WT and GFP-FAK-kinase-dead (KD) MDA-MB-231 cells (Hoechst nuclear stain, blue) reveal nucleolar GFP-FAK-WT localization (arrows). GFP-FAK-KD is primarily cytoplasmic in the confocal image. (B) Representative confocal images of MDA-MB-231 expressing GFP-FAK-WT or GFP. Scale is 50 μm. [file 13058_2015_551_MOESM4_ESM.tiff]
